# Modeling the disruptive impact of the COVID-19 pandemic on nurses’ supply and wages

**DOI:** 10.3389/fepid.2026.1631582

**Published:** 2026-06-12

**Authors:** Raffaele Vardavas, Pedro Nascimento De Lima, Lawrence Baker, Christina Crowley, Katherine Carman, Andrew M. Parker, Mahshid Abir

**Affiliations:** 1 Homeland Security Research Division, RAND Corporation, Santa Monica, CA, United States; 2 CausalPaths Analytics LLC, Los Angeles, CA, United States; 3 Alliance for Policy Research LLC, Ann Arbor, MI, United States; 4 RAND Corporation, Boston, MA, United States; 5 U.S. Securities and Exchange Commission, Los Angeles, CA, United States; 6 RAND Corporation, Pittsburgh, PA, United States

**Keywords:** behavioral economic model, COVID-19 pandemic, labor market dynamics and mechanics, nurse workforce participation, simulation model

## Abstract

**Introduction:**

The COVID-19 pandemic severely disrupted hospital nurse labor supply and wage dynamics through deteriorating working conditions, heightened occupational stress, and epidemiological pressures, exacerbating pre-existing nursing shortages. Federal relief funds were allocated to mitigate staffing shortfalls, but their effects on healthcare labor markets remain poorly characterized.

**Methods:**

We developed a behavioral economic model using a system of coupled ordinary differential equations (ODEs) to simulate hospital nurses' supply and wage dynamics at the national aggregate level during the COVID-19 pandemic. Epidemiological time-series (COVID-19 cases, hospitalizations, deaths) were provided as exogenous inputs. A perceived pandemic work burden variable—modeled as an exponential weighted moving average (EWMA) of fatigue and mental anguish proxies—drives nurse supply through a sigmoid-shaped supply surface. Travel nurse substitution dynamics and hospital relief fund eligibility constraints are incorporated. The model was calibrated using 15,000 Latin Hypercube Sampling prior runs and an Incremental Mixture Approximate Bayesian Computation (IMABC) procedure, converging to 500 posterior accepted runs.

**Results:**

The calibrated model reproduces observed dynamics for staff and travel nurse workforce participation and wages during the pandemic. Scenario analyses contrasting constrained and unconstrained hospital spending on travel nurses (governed by the parameter λ_*C*_) reveal a fundamental tension in relief fund design: unrestricted reimbursement reduces immediate staffing gaps but incentivizes excessive reliance on contract labor, while overly stringent requirements deter legitimate fund applications, particularly from rural and safety-net hospitals.

**Discussion:**

This conceptual model demonstrates that it is feasible to jointly simulate epidemiological dynamics and nurse labor market responses in a unified ODE framework. The model provides a foundation for investigating the effectiveness of pandemic relief programs and informing staffing policy for future public health emergencies, and is well-suited for embedding within robust decision-making frameworks.

## 1 Introduction

The COVID-19 pandemic has had a profound impact on nurse labor supply and wage dynamics, primarily due to deteriorating working conditions, heightened stress, and epidemiological pressures (1, 2). These factors have exacerbated pre-existing labor shortages among nurses. In response, relief funds were allocated to mitigate these shortages and reduce the financial strain on hospitals. However, the full impact of these relief funds on healthcare labor supply and wage dynamics—particularly in the context of evolving epidemiological conditions—remains unclear.

Given the critical role of hospital nurses in determining COVID-19 ICU patient outcomes (3–5), it is essential to assess how such financial interventions influence nurse labor supply and wages. To address this need, we developed a model to investigate how COVID-19 epidemiology affects the nurse labor market, including the effectiveness of relief funds aimed at alleviating staffing shortages and maintaining hospital solvency.

Despite the documented bidirectional relationship between pandemic severity and nursing workforce conditions, to our knowledge, no existing model simultaneously couples epidemiological dynamics to nurse labor market responses within a unified simulation framework. Existing integrated epidemic economic models focus on broad macroeconomic interactions, and do not account for the workforce participation dynamics of hospital nurses, a critical high-contact occupation whose availability directly shapes pandemic mortality outcomes (3).

By analyzing labor market dynamics observed during the pandemic, our model provides insights into how nurse supply and wages might respond in future pandemics or public health emergencies. This enables better preparedness and informed decision-making to manage workforce challenges under crisis conditions. Understanding the interaction between epidemiological trends and nurse labor supply is crucial for optimizing healthcare delivery and ensuring adequate staffing levels.

The conceptual model developed here represents an initial step toward building a fully integrated model (IM) capable of forecasting epidemiological indicators (e.g., incidence, prevalence), clinical outcomes (e.g., hospitalizations, mortality), and economic measures (e.g., resource utilization, costs) under a variety of future and counterfactual scenarios, including those related to COVID-19. Such an IM will improve the ability to anticipate and respond to future health crises, contributing to more effective healthcare strategies (6).

Our economic model focuses on the pandemic-induced dynamics of the nurse labor market, including demand, supply, and wage fluctuations, while distinguishing between hospital-employed staff nurses and travel nurses. Unlike long-term structural models, ours emphasizes short-term dynamics (e.g., six months to two years) that are characteristic of repeated and severe hospitalization surges during a pandemic. We demonstrate the feasibility of modeling labor market behavior in relation to epidemic dynamics (7–10).

The model explores the interaction between hospitals’ staffing decisions and nurses’ behavioral responses. It incorporates how hospitalization surges influence nurses’ willingness to continue working, as well as how hospitals adjust wages under financial constraints imposed by access to federal relief funding. The behavioral component captures nurses’ perceived pandemic-related work burden, which evolves based on experienced working conditions. These include physical fatigue, mental distress, barriers to in-person full-time work, and availability of alternative employment opportunities. Nurses’ employment decisions are modeled as a function of the trade-off between wage changes and perceived burdens. Perceptions of work burden are assumed to track COVID-19 epidemiological trends. In the absence of an explicit epidemiological model, we use historical time-series data on COVID-19 cases and hospitalizations to inform these dynamics.

The structure of this paper is as follows: Section 2 reviews prior efforts to integrate epidemiological and economic models of COVID-19, including relevant models of nurse labor supply and demand, and summarizes key policy responses. Section 3 introduces our dynamic model of nurse supply and demand during a pandemic and illustrates how relief policies may impact labor market outcomes. Sections 4, 5.2 present the calibration of our model using empirical data on nurse wages and supply and explore scenario analyses. We conclude with a discussion of the broader implications of our findings.

## 2 Brief review of COVID-19 and nurse labor market models

Empirical regression-based models have been widely used to analyze the epidemiological and economic impacts of COVID-19 (7–10). However, capturing the complex interactions between disease dynamics and economic behavior often requires integrated models (IMs) based on simulation techniques, particularly when the goal is to provide ex ante policy recommendations.

Many COVID-19 IMs combine separately developed epidemiological and economic simulation models—often rooted in fundamentally different frameworks. A common example is the integration of computable general equilibrium (CGE) models with compartmental SEIR (Susceptible, Exposed, Infectious, Removed) epidemiological models (11–14). In such models, interactions between the epidemiological and economic components occur indirectly, typically through policies that adapt to simulated outcomes. These IMs have been used to examine trade-offs between public health and economic outcomes, including national vaccination strategies and non-pharmaceutical interventions (NPIs) (15–21).

CGE models are grounded in neoclassical economic theory and typically assume rational expectations, whereby agents make decisions based on deductive reasoning and anticipated system level equilibrium conditions (22–26). Their dynamics unfold through iterative steps that reach a sequence of equilibria. However, such assumptions are problematic during pandemics, where rapid, unpredictable changes and nonlinear feedback loops undermine equilibrium-based logic (24, 27). Moreover, decision-making during COVID-19 often reflected both rational and emotional responses shaped by lived experience, not deductive optimization (28, 29). More broadly, crisis resilience also depends on social capital, institutional trust, and community support structures, which influence adaptation and recovery in ways that are not captured by standard equilibrium models (30). These limitations underscore the need for fully integrated models that explicitly couple epidemiological and economic processes, particularly in domains like the nurse labor market, where dynamic, bidirectional feedback is prominent.

A successful integration of epidemiological and economic dynamics requires models built on compatible methodological foundations. Unlike CGE models, SEIR models do not rely on equilibrium assumptions and are thus better suited for integration with economic models that accommodate disequilibrium conditions. Two primary approaches used to implement such integration are population-based models (PBMs) and agent-based models (ABMs).

PBMs adopt a system dynamics (SD) approach to model aggregated behavior using coupled ordinary differential equations (ODEs). They provide a top-down perspective that captures flows between disease compartments and are well-suited for exploring feedback mechanisms, stable system states, and policy effects at the macroscopic level.

In contrast, ABMs use a bottom-up, micro-level approach where each individual is represented as an agent interacting with others and the environment (31). ABMs account for agent heterogeneity and stochastic processes, and enable emergent macro-level patterns based on individual-level rules and interactions. Unlike PBMs, ABMs allow for the endogenous specification of behavioral rules, making them powerful tools for exploring self-organization and adaptation.

Both PBMs and ABMs can be extended to include economic decision-making, allowing behavior to adapt in response to disease dynamics. PBMs typically model behavioral adaptation implicitly at the population level, while ABMs specify individual-level rules that give rise to aggregate behavior. PBMs are more computationally efficient and easier to scale when considering many policy interventions, whereas ABMs are more expressive and can model more nuanced, targeted interventions (32, 33).

Microsimulation and ABM frameworks have been used to develop individual-level models of nurse labor supply and demand (34, 35). Notably, the Health Resources and Services Administration (HRSA) created the Health Workforce Simulation Model (HWSM), a microsimulation tool for forecasting healthcare workforce supply and demand by occupation, region, and time horizon (to 2030) (36). HWSM provides detailed projections by specialty and geographic level (e.g., county-level estimates). Meanwhile, faster, more parsimonious SD models have also been applied to assess healthcare workforce interventions (37–41).

However, none of the existing models simulate nurse labor markets during the COVID-19 pandemic using a fully integrated modeling approach. Our work addresses this gap by introducing a unified simulation framework that captures both the epidemiological and labor market dynamics specific to the hospital nursing workforce during the pandemic.

The behavioral and economic components of our model are grounded in two complementary theoretical traditions. First, the supply surface relating nurse participation to wages and pandemic work burden is anchored in compensating wage differentials,(42, 43) which imply that workers require higher pay to accept more undesirable job attributes. In our model, this relationship is represented by a sigmoid-shaped supply surface which captures how higher perceived pandemic work burden requires higher wages to sustain a given level of labor supply. Second, perceived work burden evolves according to a recency-weighted updating process which draws on adaptive expectations in labor economics.(44) In this framework, agents update beliefs about working conditions based on recent experience rather than forward-looking optimization. This departure from rational expectations is intentional, because the behavioral economics literature shows that health care workers’ decisions during crises are shaped by accumulated experience, emotional responses, and risk perception rather than deductive equilibrium reasoning (28, 29, 33). Together, these foundations place the model in an established economic tradition while allowing for the disequilibrium and behavioral realism required in pandemic labor markets. We further note that the supply dynamics also share structural features with Search-and-Matching (SaM) models in labor economics (75), particularly in the treatment of participation frictions and dynamic behavioral responses to market conditions. However, unlike SaM models, our framework does not incorporate vacancy-posting technology, Nash bargaining over wages, or a centralized matching function, as labor allocation arises from probabilistic participation governed by the sigmoid-shaped supply surface, behavioral updating, and policy constraints.

## 3 Model

The model consists of four coupled components. First, an epidemiological input layer provides exogenous weekly time series of COVID-19 hospitalizations, cases, and deaths, which drive nurse demand and perceived work burden. Second, a nurse demand and supply module translates epidemiological conditions into the quantity of nurses needed and the number of staff and travel nurses willing to work, using a sigmoid-shaped supply surface informed by compensating wage differential theory. Third, a wage dynamics model captures how staff and travel nurse wages adjust asymmetrically in response to gaps between demand and supply, with travel nurse wage growth additionally attenuated by hospitals’ exposure to relief fund eligibility constraints. Fourth, a behavioral model tracks nurses’ perceived pandemic work burden as a recency-weighted function of past epidemiological conditions. These components are coupled through a system of ordinary differential equations and solved numerically at weekly time steps. The remainder of this section describes each component in turn.

### 3.1 The economic model overview and nurse classification

Our economic model focuses on describing the supply and wage dynamics of registered nurses, taking into account their dependence on changes in demand caused by the pandemic. The model classifies registered nurses in hospitals as either staff nurses or travel nurses, without further specialization. Staff nurses are permanently employed at the hospital, while travel nurses are temporarily hired to address COVID-19 surges in hospitalization and increased demand. Although travel nurses represent a small proportion of the overall nurse labor supply, they play a significant role in providing additional supply during a pandemic, and their wages can change rapidly based on fluctuations in demand.

In our simulation model, we assume that the proportion of travel nurses hired by hospitals depends on travel nurse wages and the likelihood of obtaining federal relief funds to cover increased labor costs. It is important to avoid over-reliance on travel nurses, as it may lead to unexpectedly high labor expenses, jeopardizing hospitals’ approval of relief fund applications. Therefore, our model considers the perceived stringency of hospitals in obtaining relief funds and how it influences the desirability of relying on travel nurses as their proportion increases. By incorporating this aspect, the model allows us to explore how policies that alter the perceived stringency impact the supply and wage dynamics of staff and travel nurses.

The base economic model is inspired by previous cobweb models of nurse supply and demand dynamics (37) and adopts the assumption of sticky wages (44). We extend and couple the model to the dynamics of COVID-19 epidemiology, which are exogenous to the economic model. Thus, this analysis focuses solely on how COVID-19 surges affect nurse supply and demand and does not consider the dependence of COVID-19 hospital epidemiology on nurse supply. Treating the epidemiological dynamics as exogenous removes the bidirectional feedback between nurse supply and disease outcomes. As discussed in Section 6.1, this simplification provides a principled first step toward calibrating the more complex model that includes the bidirectional feedback.

The economic model primarily examines past supply and wage dynamics using historical weekly COVID-19 hospitalizations time-series data to capture changes in demand. The model operates at weekly time steps and considers the labor market dynamics of nurses over the past two years since the start of the pandemic. Initially, the model examines national-level nurse and travel nurse dynamics. Subsequently, the model uses the national-level outputs to inform a model specific to a particular metropolitan area of interest. In this step, the previously simulated national-level supply of travel nurses is utilized as a fixed reservoir of the maximum available travel nurses that local hospitals can employ. Additionally, the model incorporates past weekly COVID-19 hospitalizations and case data into a behavioral model, capturing nurses’ perceived pandemic-related work burden and leading to changes in the supply of staff and travel nurses.

The model includes numerous parameters and assumptions that significantly shape the dynamics. Economic parameters and assumptions involve pre-pandemic wage elasticities of supply and the functional form describing how supply changes with wages. Behavioral parameters and assumptions determine how COVID-19 epidemiology influences nurses’ perceived pandemic-related work burden , which, in turn, impacts supply when wages remain constant. Policy-related parameters and assumptions relate to the relationship between hospitals’ reliance on travel nurses and the perceived stringency in obtaining government relief funds. To assess the sensitivity of the model results, sensitivity analyses are conducted to explore the dependency on parameter values, their uncertainty ranges, and model assumptions.

Our economic model employs a system dynamics (SD) approach and is formulated using ODEs, which are numerically integrated (45). This allows the model to provide a deterministic representation of the dynamics at an aggregated population level. The SD approach is chosen because it allows us to specify the causal mechanisms driving the system’s dynamics and analyze research questions from a macroscopic perspective, which is essential for depicting a labor market impacted by a pandemic and likely operating outside of equilibrium for an extended period. Additionally, the SD approach is computationally less demanding, quicker to develop, easier to inform, and faster to run compared to individual-level agent-based models, especially when dealing with a significant number of parameters. By utilizing an SD approach for the economic model, it facilitates the task of coupling it with existing SEIR PBMs of COVID-19 transmission dynamics in future extensions. Therefore, the SD approach is well-suited for this study as it allows a rapid exploration and comparison of a wide range of scenarios and performing sensitivity analyses.

### 3.2 Nurses’ demand and supply

The participation of hospital nurses in the workforce is influenced by various factors, including supply, demand, wages, and perceived pandemic work burden . In our economic model, we take into account the dynamics of both staff and travel nurses working in hospitals. At time t, we represent the supply and demand for hospital nurses as $q_{s}(t)$ and $q_{d}(t)$ respectively. The actual number of nurses available is determined by taking the minimum between the demand and supply, given by $q(t)=\min[q_{d}(t),q_{s}(t)]$.

The total supply of nurses in the hospital, $q_{s}(t)$, is composed of two components: the staff hospital nurses denoted as $q_{H}(t)$ and the traveling nurses denoted as $q_{T}(t)$. The supply values $q_{H}(t)$ and $q_{T}(t)$ depend on a set of parameter values and dynamic variables, which we will explain in detail next.

### 3.3 Modeling nurses’ supply

When examining workforce participation in occupations like nursing, it is helpful to consider two distinct supply concepts. The first concept pertains to the proportion of available nurses, encompassing those who actively choose and desire to work as nurses. This includes all qualified individuals currently working or actively seeking nursing positions. In other words, it represents the proportion of the nursing workforce engaged and ready to contribute. On the other hand, the second concept, which might be more appropriately termed the “qualified pool” or “potential workforce,” encompasses not only those actively seeking nursing positions but also individuals who possess the necessary qualifications to work as nurses but may have temporarily chosen not to do so. This broader category includes retired nurses who could potentially return to the workforce if conditions change and those who have transitioned into different professions altogether. It is essential to acknowledge that while the second concept encompasses a wider pool, it does not account for nurses who have pursued different potentially more desirable career paths altogether, such as becoming medical doctors or researchers, etc. Our model includes these supply concepts and contributes to gaining a more comprehensive understanding of the nursing workforce, the factors influencing their choices, and the potential pool of qualified individuals available for nursing positions.

We first describe the model for the supply of hospital staff nurses, denoted as $q_{H}(w,z,n_{s},t)$, and then explain how this model is adapted for travel nurses. In our model, we assume that the supply of hospital staff nurses depends on three factors, whose quantities can change dynamically: (i) their average wage w(t), (ii) their perceived pandemic work burden z(t), and (iii) the total qualified pool of available registered nurses in the system $n_{s}(t)$, which includes those who can work in hospitals but choose not to.

**Figure 1:**
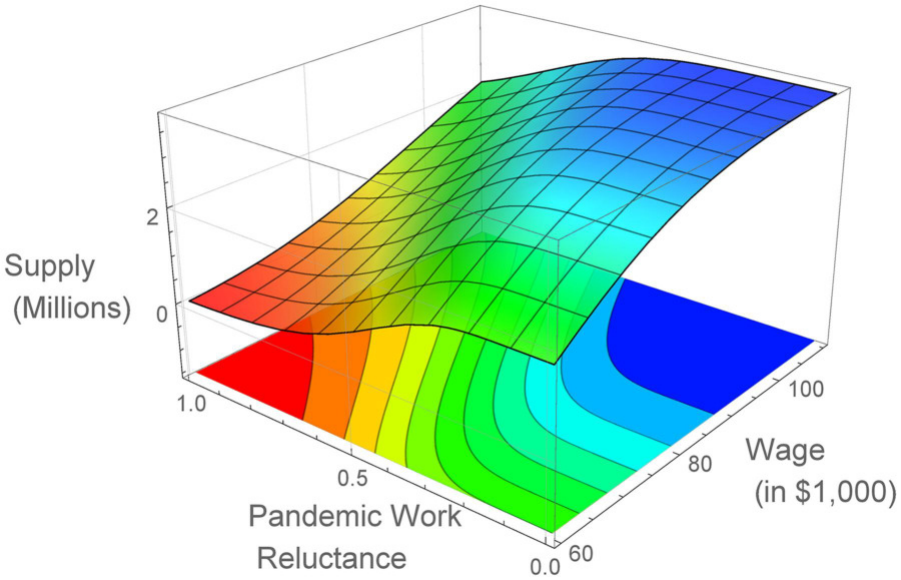
Pandemic-shaped Supply surface.The two sets of orthogonal grid lines in the S-surface plot respectively track equi-distant cross-sectional profiles of the S-curves for constant w and z. The curves of similar color track the contour-lines of equal supply, and provide the compensating wage differential which can be used to translate the perceived pandemic work burden measure to an equivalent dollar amount.

We make the assumption that the supply of a workforce can be described by a sigmoidal function denoted as $S_{\theta }(n_{s},w,z)$, which defines a surface over the w-z plane as illustrated in Figure 1. The subscript θ represents the set of parameter values that determine the specific shape of the sigmoidal function.

To specify our surface, we employ the cumulative distribution functions (CDF) of a log-normal distribution given by the equation:

$$S_{\theta }(n_{s},w,z)=\frac{1}{2}\;n_{s}\left\{1+\text{erf}[s_{s}[\ln(w)-\ln(m_{s}(z))]]\right\}.$$ (1)

Our sigmoidal function is based on using the error function (erf(x)). In economics, sigmoid functions, such as probability CDF utilizing the error function, have been used to describe changes in utilities (46). Equation 1, represents the surface that determines the supply of nurses. The curve $S_{\theta }(n_{s},w,0)$ corresponds to the pre-pandemic supply dependence on wages. This is because by definition the perceived pandemic work burden in the pre-pandemic period is zero (i.e., z=0). The shape of this curve depends on the parameter $s_{s}$, which controls its steepness with changing wages, and the wage-rate $m_{s}(0)$, representing the level at which the supply is expected to be half of $n_{s}$.

As shown in the contour plot of the S-surface in Figure 1, and in line with the theory of compensating-wage differential, it is necessary for wages to increase as perceived pandemic work burden rises in order to maintain the supply of nurses. Consequently, the wage-rate $m_{s}(z)$, where supply is expected to be half of $n_{s}$, also increases with the level of perceived pandemic work burden (z). We assume that $m_{s}(z)$ follows a similar sigmoid function and can be expressed as:

$$m_{s}(z)=m_{s}(0)\left\{1+\frac{1}{2}\;\left[1+\text{erf}[s_{z}[\ln(z)-\ln(m_{z})]]\right]\right\}.$$ (2)

For a fixed wage rate, the parameter $m_{z}$ represents the value of perceived pandemic work burden at which supply is half of its pre-pandemic level, while the parameter $s_{z}$ controls the steepness of the curve along the z direction. To simplify the model, we assume that $m_{z}$ and $s_{z}$ are constant values that do not change with wages. Therefore, the parameter set θ is defined by $m_{s}(0)$, $m_{z}$, $s_{s}$, and $s_{z}$. Our model specifies two different parameter sets $\theta_{H}$ and $\theta_{T}$ that respectively inform the supply of staff hospital nurses $q_{H}(t)$ and the traveling nurses $q_{T}(t)$.

To describe the supply dynamics of travel nurses, we utilize a similar S-curve function as used for staff hospital nurses. This function is defined by Equations 1, 2, but with different parameter values for $n_{s}$, $s_{s}$, $m_{s}(0)$, $s_{z}$, and $m_{z}$. We determine these parameter values through a combination of wage elasticity estimates found in the literature and model calibration methods. Further details regarding the determination of parameter values for staff and travel nurses will be discussed in Section 4.

Equations 1, 2 provide the equilibrium supply of nurses when $n_{s}$, w, and z are fixed. However, since each of these variables changes over time t, the dynamics of supply are described by the following partial differential equation (PDE) (Equation 3):

$$\frac{dS_{\theta }}{dt}=\frac{\partial S_{\theta }}{\partial t}+\frac{\partial S_{\theta }}{\partial n_{s}}\frac{dn_{s}}{dt}+\frac{\partial S_{\theta }}{\partial w}\frac{dw}{dt}+\frac{\partial S_{\theta }}{\partial z}\frac{dz}{dt}.$$ (3)

In this PDE, the first term $\partial S_{\theta }/\partial t$ represents the long-term supply trend, independent of changes in wages, pandemic-related outcomes, and the qualified pool of registered nurses available in the system. The second term, the product of $\partial S_{\theta }/\partial n_{s}$ and $dn_{s}/dt$, describes the long-term supply dynamics of the qualified pool of registered nurses in the system, accounting for changes due to recruitment and expansion policies, as well as contractions such as nurses permanently retiring. However, since our focus is on the short-term dynamics of staff nurses during the pandemic, we assume that $\partial S_{\theta }/\partial t$ and $dn_{s}/dt$ are both zero. Therefore, we retain only the last two terms of the PDE, which describe the short-term dynamics of the supply of staff nurses as wages and perceived pandemic work burden change. These terms depend on the derivatives $\partial S_{\theta }/\partial w$ and $\partial S_{\theta }/\partial z$, respectively.

To derive the expressions for these derivatives, we take the respective derivatives of the S-surface given by Equations 1, 2. Since $S_{\theta }(n_{s},w,z)$ represents a scaled log-normal cumulative distribution function (CDF), its derivative corresponds to a scaled log-normal probability density function (PDF). The expressions for the derivatives are as follows (Equations 4 and 5):

$$\frac{\partial S_{\theta }}{\partial w}=\frac{n_{s}s_{s}\exp\left\{-s_{s}^{2}[\ln w-\ln m_{s}(z){]}^{2}\right\}}{w\sqrt{\pi }}.$$ (4)

The expression for $\partial S_{\theta }/\partial z$ is obtained by taking the product of $\partial S_{\theta }/\partial m_{s}(z)$ and $\partial m_{s}(z)/\partial z$, which are given by:

$$\begin{array}{cc} \frac{\partial S_{\theta }}{\partial m_{s}} & \;\;\;=-\frac{n_{s}s_{s}\exp\left\{-s_{s}^{2}[\ln w-\ln m_{s}(z){]}^{2}\right\}}{m_{s}\sqrt{\pi }},\\ \frac{\partial m_{s}}{\partial z} & \;\;\;\;\;\;\;\;\;\;\;\;\;=\frac{m_{s}(z)s_{z}\exp\left\{-s_{z}^{2}[\ln m_{z}-\ln z{]}^{2}\right\}}{z\sqrt{\pi }}. \end{array}$$ (5)

To fully describe the dynamics of supply, we also need the differential equations governing the wage and perceived pandemic work burden dynamics, namely dw/dt and dz/dt. These differential equations couple the supply dynamics with the changing demand for staff hospital nurses and the behavioral model for perceived pandemic work burden, respectively. These models depend on observed epidemiological outcomes related to COVID-19, including the number of cases, hospitalizations, and deaths. The details of these models will be introduced in sections related to wage dynamics and perceived pandemic work burden dynamics.

### 3.4 Interpretation of the supply surface

Wage rates in a specific occupation are influenced by factors such as the perceived unpleasantness, risk, or other undesirable attributes associated with the job. Compensating wage differential analyses help quantify the additional monetary compensation required to motivate individuals to accept an unpleasant job compared to alternative job options. Under normal pre-pandemic conditions, the perception of unpleasantness and risk remains relatively stable or changes gradually over time. While static risk models may be suitable for risks that do not significantly fluctuate within short timeframes, this approach is not be applicable during a pandemic.

During the COVID-19 pandemic, the willingness to work as a nurse varied throughout its duration. In our model, we assume that the pre-pandemic considerations regarding the unpleasantness of nursing remain constant and incorporate them into the supply’s dependence on wages. To account for the additional unpleasantness and risk perceived by nurses due to the direct and indirect effects of the pandemic, we introduce the measure z as perceived pandemic-related burden to work. Alternatively, we could have defined z as an absolute measure of risk and unpleasantness. For example, challenges in finding adequate childcare for nurses with young children existed before the pandemic, which significantly contributes to the absolute measure of occupational unpleasantness, especially considering the predominantly female nursing workforce. However, in our measure of z, we only consider the extra difficulties faced by nurses in finding or increasing hours of childcare due to the pandemic-induced over-hours requirement at work.

Moreover, it is important to note that the S-surface represents an aggregated average of wage preferences and perceived pandemic-related work burden across a diverse population of nurses. Each nurse can be assumed to have their own unique S-shaped surface over these dimensions, which collectively forms the curve used in our model at the population level. In this initial version of our model, we simplify by assuming a homogeneous nurse population, without stratification based on age, years of experience, marital status, poverty level, or other critical demographic variables that may influence the shape of the S-surface. However, it is crucial to acknowledge that wage preferences and perceived pandemic burden differ among various nurse population groups, leading to distinct decisions over time. These decisions, in turn, reshape the demographic composition of nurses employed by hospitals, resulting in changes to the S-surface’s shape over time.

### 3.5 Wage dynamics

In our model, the wages of hospital staff are influenced by the disparity between demand and supply, and they are assumed to be inflexible, resulting in asymmetric adjustments. When wages are below the market-clearing rate, hospitals need to increase wages to fill vacancies. Conversely, when wages exceed the market-clearing rate, hospitals may want to reduce them, but contractual arrangements prevent such adjustments. The rate of change of hospital staff wages, denoted as $w_{H}(t)$, is described by the equation (Equation 6):

$$\frac{dw_{H}}{dt}=[(\alpha_{\mathrm{Hd}}-\alpha_{\mathrm{Hs}})H(q_{d}-q_{s})+\alpha_{\mathrm{Hs}}]\cdot (q_{d}-q_{s}),$$ (6)

Here, H(x) represents the Heaviside step function, which we approximate using an inverse logistic function for integration by the numerical solver in our model implementation. The α parameters, specified as a dollar rate per nurse, determine how the wage gap between demand and supply affects wage changes. Wages increase more rapidly at a rate $\alpha_{\mathrm{Hd}}$ when there is excess demand for nurses, while they decrease at a rate $\alpha_{\mathrm{Hs}}$ when there is an equivalent excess supply. Notably, the total supply $q_{s}$ of both staff and travel nurses is used to compute the gap between demand and supply, rather than relying solely on the supply of hospital staff nurses.

We also assume that hospitals employ travel nurses to manage COVID-19 surges in hospitalizations and increased demand. However, the proportion of travel nurses hired during these surges depends on travel nurse wages and the likelihood of obtaining federal relief funds to cover increased labor costs. If labor expenses exceed expectations due to an excessive reliance on travel nurses, the application for relief funds may be partially approved or rejected. To capture this dynamic, we use a similar equation to that of hospital staff nurses to model the rate of change of travel nurse wages, denoted as $w_{T}(t)$, but with an additional multiplicative scaling term. This equation is given by (Equation 7):

$$\frac{dw_{T}}{dt}=[(\alpha_{\mathrm{Td}}-\alpha_{\mathrm{Ts}})H(q_{d}-q_{s})+\alpha_{\mathrm{Ts}}]\cdot (q_{d}-q_{s})\cdot \exp[-\lambda_{C}w_{T}(t)q_{T}(t)/C(t)].$$ (7)

For travel nurses, we use different α parameters, reflecting their distinct values compared to hospital staff nurses. We anticipate that the difference between $\alpha_{\mathrm{Td}}$ and $\alpha_{\mathrm{Ts}}$ is smaller than the corresponding difference for hospital staff nurses, as wages for travel nurses are more flexible and less rigid. Furthermore, we expect $\alpha_{\mathrm{Td}}\gg \alpha_{\mathrm{Hd}}$ since travel nurse wages are more responsive to changes in increased demand. The term $C(t)=w_{S}(t)q_{S}(t)+w_{T}(t)q_{T}(t)$ represents the total cost of nurses for hospitals. Through the multiplicative scaling term $\exp[-\lambda_{C}w_{T}(t)q_{T}(t)/C(t)]$, the increase in travel nurses’ wages is attenuated as the proportion of hospitals’ labor expenses allocated to travel nurses rises. The tunable constant parameter $\lambda_{C}$ controls the dynamics of this scaling.

### 3.6 The perceived pandemic-induced work burden

In the introduction, we emphasized several pandemic-related factors that have made working as a hospital nurse more challenging and risky. These factors include fatigue from repeated COVID-19 hospitalization surges, the increased risk of contracting COVID-19 in the workplace, unclear or impracticable quarantining guidelines and mandates, increased proximity to preventable deaths, shortages in personal protective equipment during surges, and an increased nurse-to-patient ratio during surges. Working during a pandemic is objectively more difficult than working under normal conditions. The objective of our model is to incorporate these factors by introducing a concise measure of perceived pandemic-induced work burden. To capture these factors, our model defines a work burden measure, denoted as z(t), which is a function of observed epidemiological time-series $X_{t_{0},\ldots ,t_{i}}$ and a set of parameters. The assumption we make is that the observed epidemiological time-series provide proxies to how perceived pandemic-induced work burden component factors have changed during the pandemic. It is important to note that precisely capturing and predicting this concept is inherently challenging, and this exploratory study does not achieve this. However, we propose and compute a potential measure of perceived pandemic-induced work burden for this conceptual model. We assume that perceived pandemic work burden can be represented by the equation (Equation 8):

$$z(t)=(1-s)\sum_{m=1}^{t}s^{t-m}\Delta (m),$$ (8)

Here, s is a discount parameter, and m represents time in weekly units. The equation represents an exponential weighted moving average (EWMA) process over Δ(t), which measures nurses’ past weekly evaluations of the pandemic conditions affecting their perceived pandemic work burden. The range of Δ(t) is between 0 and 1, and the term (1−s) in the equation serves as a normalizing factor. The discount parameter s can be transformed into a half-life duration τ, expressed in weeks, using the formula −ln⁡(2)/ln⁡(s). The half-life represents the duration for past evaluations to contribute half as much to the perceived pandemic work burden measure.

In our study, evaluations depend on the weighted sum of two components, expressed as:

$$\Delta (t)=\beta_{F}\phi_{F}(t)+\beta_{M}\phi_{M}(t),$$ (9)

Here, the coefficients β represent weights that sum up to one. Each of the components ϕ is evaluated weekly. Specifically, $\phi_{F}$ measures nurses’ pandemic-related fatigue, while $\phi_{M}$ quantifies their mental anguish. We assume that fatigue is proportional to the number of COVID-19 hospitalizations, while mental anguish is proportional to the number of COVID-19 deaths. However, these measures are scaled to mainly vary between 0 and 1. For instance, when COVID-19 hospitalizations are zero, fatigue levels are at pre-pandemic levels, and $\phi_{F}$ does not contribute to the perceived pandemic work burden measure. On the other hand, when $\phi_{F}$ is at or above one, fatigue is critically high due to a significant surge in COVID-19 hospitalizations, indicating an unsustainable workload for the hospital. Similarly, when the value of $\phi_{M}$ is zero, nurses’ mental anguish is at pre-pandemic levels due to the absence of observed COVID-19 deaths. When $\phi_{M}$ is at or above one, it signifies high and critically unsustainable mental anguish.

Additional terms could be considered in Equation 9 defining Δ(t). For example, a term $\beta_{C}\phi_{C}(t)$ could be included, where $\phi_{C}$ measures additional barriers for nurses to get to work at hospitals during the pandemic due to imposed social distancing policies affecting them, their family and loved ones. In this case $\phi_{C}$ would be linked to COVID-19 case counts or the non-pharmaceutical interventions policy level.

## 4 Informing the model

Our model relies on publicly available datasets to inform the initial conditions of our simulation models and calibration targets. These datasets provide information on various aspects, such as nurses’ supply and wage dynamics, epidemiological parameters, and time series related to nurses’ demand and perceived pandemic work burden. To obtain these data, we accessed national and regional healthcare wage and employment time series from the Bureau of Labor Statistics (BLS) (47–49). Additionally, we constructed wage and employment data for travel nurses using historical trends from contract nursing agencies, encompassing both pre-pandemic and pandemic periods (50–52), building upon previous analyses (53).

For our epidemiological inputs, we utilized daily state-level data on COVID-19 hospitalizations and deaths from the COVID Tracking Project (54). We also monitored non-pharmaceutical interventions (NPIs) implemented at the state level through databases maintained by Oxford University and Boston University (55, 56).

Table 1 provides an overview of the model parameters used in our analysis. We will refer to these parameter values throughout the presentation as we explain how they informed our model.

**Table 1:** Model input values and ranges used for initialization and calibration.

Input	Description	Value	Min	Max	Source
$n_{a}$	All registered nurses employed (persons)	$3.53\times 10^{6}$	$2.75\times 10^{6}$	$3.84\times 10^{6}$	(37)
$w_{a}$	Annual wage, all registered nurses (USD/year)	$8.96\times 10^{4}$	–	–	(37)
$\epsilon_{s,a}$	Short-run labor supply elasticity, all registered nurses	0.644	–	–	(37)
$w_{h}$	Annual wage, hospital registered nurses (USD/year)	$8.96\times 10^{4}$	$8.00\times 10^{4}$	$1.00\times 10^{6}$	(36)
$n_{t}$	Travel registered nurses employed (persons)	$6.30\times 10^{4}$	0	$8.40\times 10^{4}$	(36)
$w_{t}$	Annual wage, travel registered nurses (USD/year)	$2.50\times 10^{5}$	$8.96\times 10^{4}$	$5.20\times 10^{5}$	(36)
$\epsilon_{s,t}$	Short-run labor supply elasticity, travel registered nurses	6.0	–	–	(53)
$n_{ti}$	Travel ICU registered nurses employed (persons)	$2.52\times 10^{4}$	0	$3.36\times 10^{4}$	(37)

The “Value” column reports the baseline estimate used in the model. Where applicable, the “Min” and “Max” columns define the range over which parameters are varied in sensitivity analysis or calibration. Entries marked with “–” indicate fixed inputs that are not varied.

### 4.1 Informing the prepandemic supply function

We begin by utilizing the data from the Bureau of Labor Statistics (BLS) to estimate the parameter values for $n_{s}$, $m_{s}(0)$, and $s_{s}$, which define the S-curve of hospital staff nurses in our national-level model. According to the BLS data for 2020, there were 3.53 million registered nurses (RNs). We use this value as our estimate for the pre-pandemic supply of hospital staff nurses. We use the 2020 nursing survey to estimate a lower bound value of 2.75 million by reducing this supply by the estimated number nurses that are willing to retire or find a different occupation in the next five years. Similarly, using the 2020 nursing survey we estimate an upper bound value of 3.84 million by increasing the supply by adding 50% of the estimated pool of nurses that have retired, 50% of the unemployed nurses seeking non-nursing occupations and 100% of unemployed nurses seeking nursing occupations. In addition, we use this upper bound as our estimate of the total qualified pool of nurses that could be drawn upon.

The average salary of RNs was $89,600, and the short-term wage elasticity of supply was estimated to be 0.644 (37). When z=0 and $w=8.96\times 10^{5}$, the corresponding supply, $q_{s}$, is $3.53\times 10^{6}$. Assuming that the pre-pandemic supply follows the sigmoid function $S(n_{s},w,0)$ as defined in Equation 1, we have (Equation 10):

$$S(n_{s},w,0)=\frac{1}{2}\;n_{s}\left[1+\mathrm{erf}(\tilde{w})\right],$$ (10)

where $\tilde{w}=s_{s}[\ln(w)-\ln(m_{s}(0))]$. By knowing the values of $n_{s}$ and $q_{s}$ when $w=8.96\times 10^{5}$, we can estimate the value of $\tilde{w}$. Next, we use the pre-pandemic wage elasticity of supply, $\epsilon_{s}$, to estimate the parameter values of $m_{s}(0)$ and $s_{s}$. The local elasticity of supply is defined as (Equation 11):

$$\epsilon_{s}=\frac{\left(q_{s}^{-1}\partial q_{s}\right)}{\left(w^{-1}\partial w\right)}.$$ (11)

This equation can be rearranged as (Equation 12):

$$w\partial q_{s}/\partial w=\epsilon_{s}q_{s}.$$ (12)

By differentiating $S_{\theta }(n_{s},w,0)$ with respect to w and evaluating it at the pre-pandemic values of wage and employed staff nurses, we have (Equation 13):

$$w\frac{\partial S_{\theta }(n_{s},w,0)}{\partial w}=\frac{n_{s}s_{s}\;e^{-{\tilde{w}}^{2}}}{\sqrt{\pi }}.$$ (13)

Since the local value of $w\frac{\partial S_{\theta }}{\partial w}$ is equal to $w\frac{\partial q_{s}}{\partial w}$, we can equate $\epsilon_{s}q_{s}$ to $n_{s}s_{s}e^{-{\tilde{w}}^{2}}/\sqrt{\pi }$. Using our estimated value for $\tilde{w}$, we can determine the values of $s_{s}$ and $m_{s}(0)$. The expressions for these parameters are given by (Equation 14):

$$\begin{array}{cc} \tilde{w} & ={\mathrm{erf}}^{-1}\left(\frac{2q_{s}}{n_{s}}-1\right),\\ s_{s} & =\frac{\epsilon_{s}q_{s}\sqrt{\pi }e^{{\tilde{w}}^{2}}}{n_{s}},\\ m_{s} & =we^{-\tilde{w}/s_{s}}. \end{array}$$ (14)

Hence, we obtain $m_{s}(0)=\$62,827$ and $s_{s}=2.78$, which are assumed characterize the pre-pandemic supply curve.

Moving on to the parameters $m_{z}$ and $s_{z}$, which define the shape of the supply S-surface in the z direction, estimating these values is more challenging and depends on the values used to compute the pandemic work burden , z. To address this, we start with a wide range of uncertainty for these parameters and use model calibration to narrow down their values. However, to simplify the calibration process, we make the assumption that the normalized gradient at $z=m_{z}$ is a constant and independent of wage. This assumption fixes the value of $s_{z}$ relative to $m_{z}$. In our S-surface example presented in Figure 1, we assume that in the absence of changes in wages, the supply decreases by 50% when z=0.9 for the prepandemic disease-free state, leading to $m_{z}=0.9$. Additionally, we set the constant gradient equal to one, resulting in $s_{z}=m_{z}\sqrt{\pi }$. Table 2 summarizes the computed input parameters for the hospital RN.

**Table 2:** Estimated parameter values for the hospital RN.

Population	w	ϵ	$q_{s}$	$n_{s}$	$\tilde{w}$	$s_{s}$	$m_{s}$
All RNs	89,600	0.644	3,530,544	3,842,553	0.988	2.783	62,826

We repeat the same process for travel nurses, who have an independent S-curve of the same form. The key distinction between travel nurses and staff nurses lies in the elasticity of supply for travel nurses, which is roughly ten times higher (53). This means that minor variations in wages can lead to substantial changes in the supply of travel nurses. Travel nurses work under short-term contracts and anticipate working in various locations, and so encounter fewer obstacles to relocation compared to staff nurses. Consequently, travel nurses are more able to move to the hospital that provides the highest wage offer.

### 4.2 Demand

In our model, we assume that demand for nurses is proportional to the demand for beds, where beds can be occupied by those with COVID-19 or those without COVID-19. The primary source for demand data was the HHS COVID-19 Reported Patient Impact and Hospital Capacity by Facility survey (76), which reports weekly counts of COVID and non-COVID bed occupancy. However, data from the HHS is unreliable during the first few months of the pandemic, because relatively few hospitals reported. For the period when fewer than 90% of hospitals were reporting, we supplemented the HHS data using estimates of total hospital admissions from the Kasier Family Foundation (57) and estimates of COVID admissions the Carlson School of Management, University of Minnesota (77). To convert between admissions (flow) and occupancy (stock) we assume that COVID-19 admissions have a length of stay of 10 days, and non-COVID-19 admissions have a length of stay of 4.5 days. We then scale the HHS/KFF data such that the timeseries is continuous at the point we switch to HHS data.

We convert bed demand into nurse demand using a scalar—assuming a bed-to-nurse ratio of 0.23. This ratio was obtained by dividing pre-pandemic bed demand by pre-pandemic nurse employment. While this ratio may seem low, this accounts for the need for multiple shifts of nurses for each bed and outpatient nurses who are not directly tied to beds (but who we assume have demand proportional to bed demand).

In our model, capacity is represented on a scale from 0 to 1, where a capacity of 0 corresponds to hospital bed usage at the pre-pandemic level (80%), and a capacity of 1 indicates the utilization of the total bed capacity (100%). When the capacity is above 80% (greater than 0), it signifies that the hospital is under stress. The capacity measure specifically captures hospital bed utilization ranging from 80% to 100%.

If there is a week during which the hospital does not admit any new patients, the capacity measure is reset to its baseline value of zero. To calculate the capacity parameter, we multiply the 20% excess (the difference between the pre-pandemic and pandemic capacity) by 5. This multiplication converts the additional bed capacity to a scale between 0 and 100%. Therefore, the capacity is computed as the weekly number of COVID-19 hospital admissions divided by the total number of hospital beds, divided by 0.2, and then multiplied by the ratio of the average length of stay for COVID-19 patients to non-COVID-19 patients.

### 4.3 Perceived pandemic work burden parameters

**Figure 2:**
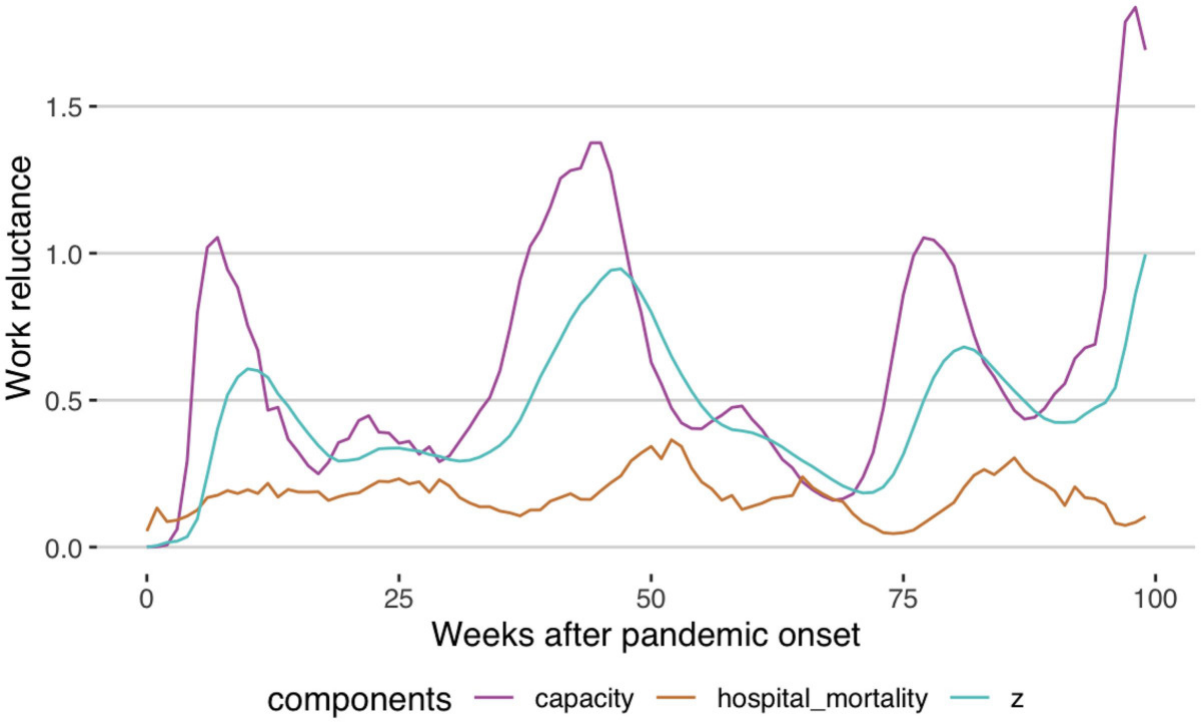
Illustration of how the perceived pandemic work burden z shown as a blue curve follows the changes in capacity and COVID-19 mortality with a lagged time.

Figure 2 shows the time-series of $\phi_{M}$ and $\phi_{F}$. Here, $\phi_{M}$ is given by the time series of COVID-19 deaths to COVID-19 hospitalizations. Component $\phi_{F}$ is given by scaling COVID-19 hospitalizations on the assumption that in the DF hospital capacity operates at 80% and that COVID-19 patients stay in the hospital on average for 10 days as opposed to other patients in the DF state which on average stayed in the hospital for 4.5 days. For our preliminary analysis, we further assume that $\beta_{M}=0.3$ and $\beta_{F}=0.7$. Hence we do not consider the effects of $\phi_{C,t}$. Our discount parameter s is chosen to be equal to 0.7 which translates to a half-life of −ln⁡(2)/ln⁡(s)∼2 weeks. The choice of this value represents a relatively short memory window; the sensitivity of model outputs to this parameter is discussed further in Section 6.1.

The values of the baseline weights were informed by analyses of longitudinal national surveys of nurses conducted during the pandemic (58). These surveys asked nurses who expressed intention to leave their positions within one year to rate the importance of 15 factors and 24 barriers to continued employment. Categorizing these factors into fatigue- and distress-related components revealed that fatigue was the dominant driver, accounting for approximately 50% of stated importance, while distress accounted for approximately 30%, with an estimated accuracy range of ±15% around each value (59). These survey-derived weights informed the baseline parameterization in the present study and in a subsequent application of the model to Virginia’s healthcare workforce (59). The sensitivity of model outputs to the behavioral parameters—including the fatigue weight, the distress-independent fixed proportion, and the EWMA discount parameter—was characterized in that application, where fatigue weighting, initial demand, and the discount half-life were identified as the highest-leverage parameters governing work reluctance dynamics. A dedicated sensitivity analysis of the β weights in the present national-level model is an acknowledged limitation and a priority for future work (see Section 6.1).

## 5 Results

### 5.1 Example simulation runs

**Figure 3:**
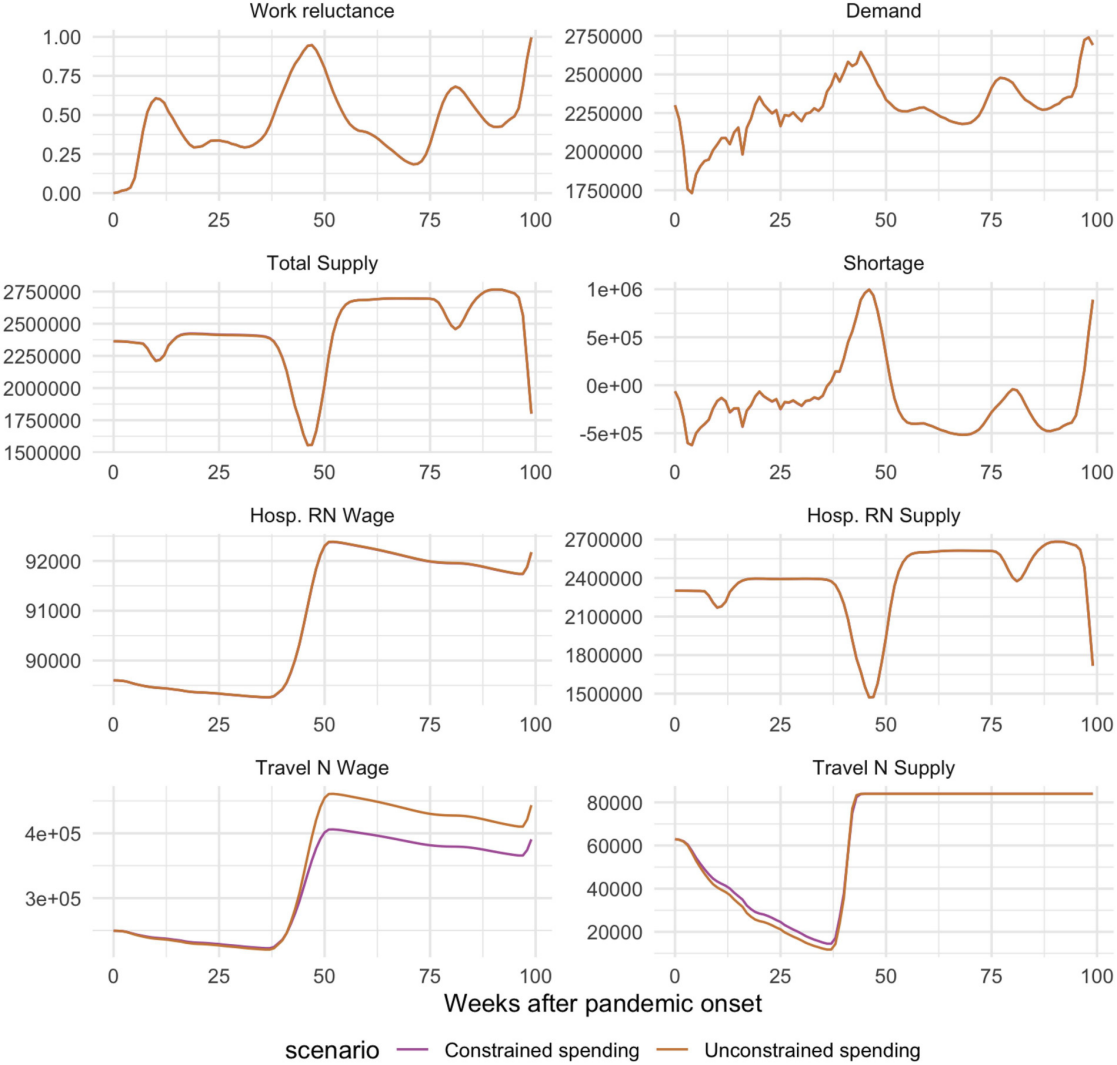
Illustrative plot of state variable dynamics generated by our model for two example cases, distinguished solely by the parameter $\lambda_{C}$ governing hospitals’ inclination to utilize travel nurses.

To provide an illustrative depiction of model outputs, we examine two distinct case runs. In the first case, we set $\lambda_{C}$ to a value of one. In the second case, we use a value for $\lambda_{C}$ that is three times larger, representing a scenario where hospitals exhibit greater reluctance to rely on travel nurses due to concerns about potential limitations in government relief fund reimbursement for pandemic-related costs. These scenarios are termed ”unconstrained” and ”constrained” spending, respectively. Figure 3 displays the model outputs for these two case runs, arranged for easy visual comparison. We see how $\lambda_{C}$ affects the dynamics of travel nurse compensation.

### 5.2 Model calibration

In Section 5.1, we examined a specific pair of case runs by varying a single parameter. However, this limited perspective does not capture the full spectrum of dynamics our model can generate. Like many other simulation models of complex dynamic systems, our model relies on simplifying assumptions and parameter values that are challenging to estimate from available data. This results in considerable uncertainty in the model inputs. To comprehensively understand the model’s behavior and align it with observed data, calibration is crucial to adjust the model parameters.

Our calibration process involves generating 15,000 distinct and independent parameter combinations, all falling within the defined uncertainty range for case runs. Each of these cases is simulated using our model. This selection of parameter values and the number of case runs define our experimental design. By simulating a consistent nurse population across a wide range of parameter values and policy settings, we account for the inherent variability associated with parameter shifts. This comprehensive approach includes a substantial number of case runs, which reflect the diverse variation in model parameter values. This consideration is essential for capturing the inherent variability resulting from parameter changes. The trajectories of output state variables related to supply and wages are then compared to our calibration time-series targets.

Our calibration targets consisted of two time series: (i) monthly national-level staff registered nurse employment and average wages, obtained from the Bureau of Labor Statistics (BLS) Current Employment Statistics and Occupational Employment and Wage Statistics programs (47–49), and (ii) travel nurse employment counts and average compensation, reconstructed from historical data published by contract nursing agencies spanning both pre-pandemic and pandemic periods (50–52), building on prior analyses (53).

The uncertainty ranges sampled by LHS were derived from three sources, summarized in Table 1. First, literature-based estimates informed pre-pandemic supply parameters: the wage elasticity of staff nurse supply was taken from (37) and the substantially higher elasticity of travel nurses from (53), with uncertainty ranges reflecting the spread of estimates across studies. Second, pre-pandemic employment and wage initial conditions were derived from BLS and HRSA administrative data (36, 49), with bounds defined by observed inter-year variation. Third, behavioral parameters governing perceived work burden, including the component weights $\beta_{F}$ and $\beta_{M}$, the EWMA discount half-life, and the supply surface parameters in the z-direction, were assigned prior ranges informed by analyses of longitudinal nurse surveys (58) and by the analytical procedure described in a subsequent application of this framework (59). Because direct empirical estimates of these behavioral parameters are unavailable, their ranges were intentionally wide to allow the calibration procedure to identify combinations consistent with observed workforce dynamics rather than to confirm pre-specified values.

We used the Incremental Mixture Approximate Bayesian Computation (IMABC) procedure for our calibration (60). IMABC is a statistical method that approximates the posterior distribution of a complex model when the likelihood function is intractable or computationally expensive to evaluate. It is an extension of the Approximate Bayesian Computation (ABC) method that employs the Latin hypercube sampling (LHS) technique to improve the efficiency of the algorithm. LHS is a statistical sampling method that generates samples from a distribution by dividing the range of each variable into equally probable intervals and randomly selecting one value from each interval. This technique ensures that the samples are representative of the entire parameter space and reduces the number of samples required to obtain accurate results. Commencing with 15,000 initial case runs representing our priors, our calibration procedure employs an iterative approach to select and regenerate samples from these priors. This iterative process converges towards our calibration targets, resulting in a posterior set of 500 case runs that constitute our calibrated runs.

The final IMABC tolerance thresholds used as acceptance criteria were approximately $645,000 for staff nurse employment, $43,000 for travel nurse employment,$24,000 for staff nurse wages, and$45,000 for travel nurse compensation, corresponding to roughly 2.6 empirical standard errors of each target time-series. Table 1 summarizes the central values and ranges used in the present study, and Table 3 provides the full prior distributions for all free parameters used in the LHS sampling.

**Table 3:** Prior distributions for freely sampled parameters in the Latin Hypercube Sampling step of the national-level IMABC calibration.

Parameter	Description	Prior	Min	Max
$\alpha_{\mathrm{Hd}}$	Demand-side wage adjustment rate, staff nurses	U	1,000	3,500
$\alpha_{\mathrm{Hs}}$	Supply-side wage adjustment rate, staff nurses	U	55	420
$\alpha_{\mathrm{Td}}$	Demand-side wage adjustment rate, travel nurses	U	1,500	4,500
$\alpha_{\mathrm{Ts}}$	Supply-side wage adjustment rate, travel nurses	U	150	450
$\lambda_{C}$	Travel nurse expenditure attenuation	U	0	3
$m_{z}$	S-surface z-direction location, staff nurses	U	1.0	2.0
$m_{z,T}$	S-surface z-direction location, travel nurses	TN(3.0,0.2)	2.0	4.0
$\epsilon_{s,H}$	Short-run wage elasticity, staff nurses	TN(0.644,0.1)	0.40	0.80
$\epsilon_{s,T}$	Short-run wage elasticity, travel nurses	TN(6.0,1.0)	4.0	8.0
$\beta_{F}$	Work burden weight—fatigue component ($\phi_{F}$)	U	0	0.5
$\beta_{M}$	Work burden weight—mortality component ($\phi_{M}$)	U	0	0.5

U denotes a uniform distribution over the range specified in the Min/Max columns; TN(μ,σ) denotes a normal distribution with mean μ and standard deviation σ, truncated to the range specified in the Min/Max columns. Fixed initial-condition parameters ($n_{H}$, $n_{T}$, $w_{H}$, $q_{H}$, $w_{T}$, $q_{T}$) are set from BLS and HRSA administrative data and listed in Table 1.

**Figure 4:**
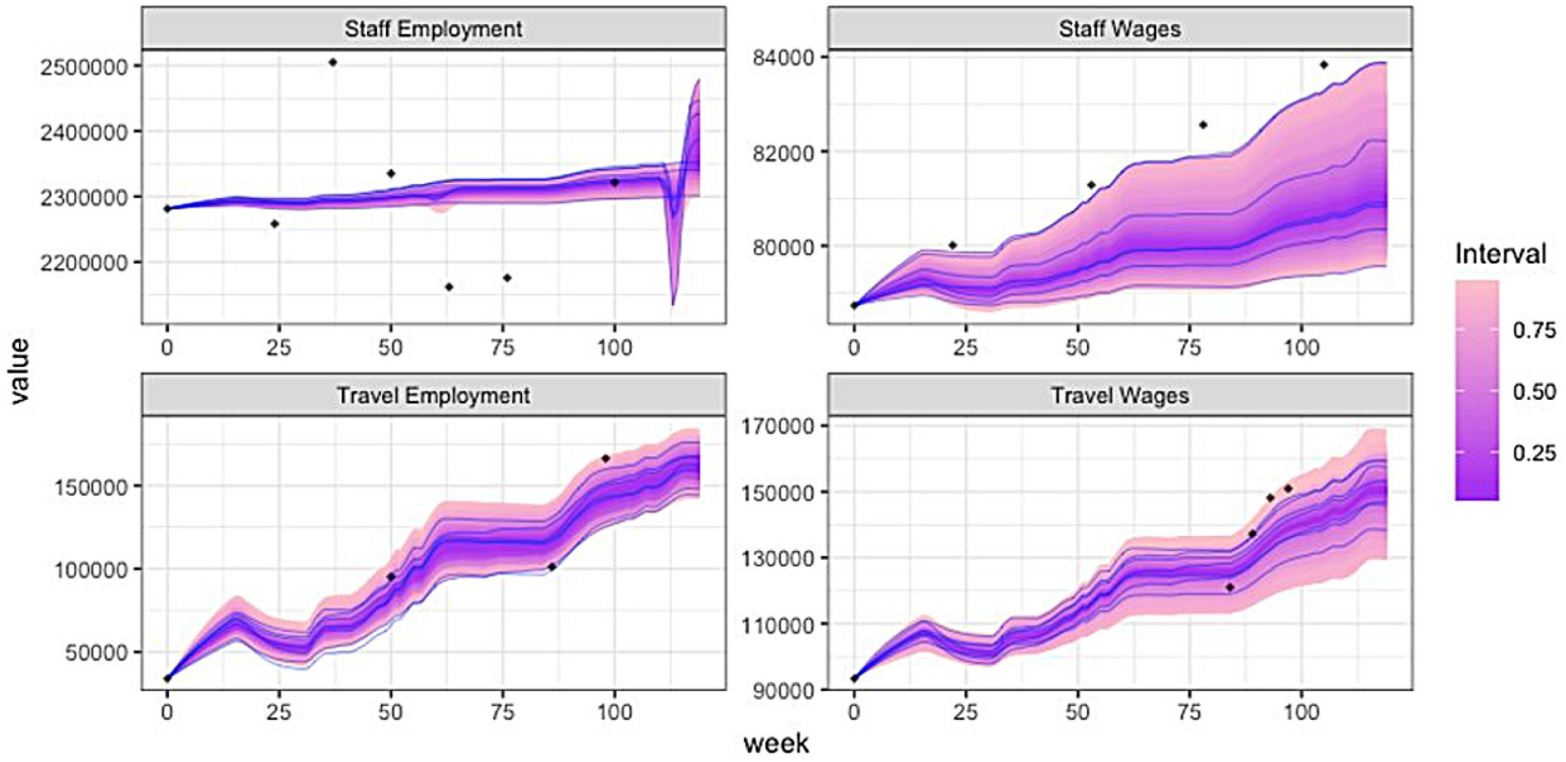
Illustrative plot of the calibration results, showcasing distributions of model runs and sample trajectories for staff and travel nurse counts, wages, and compensation, with calibration targets represented as black points.

Displayed in Figure 4 are the posterior outcomes of our calibration procedure. The visualization comprises four panels, each depicting the count of staff nurses employed along with their corresponding wages, as well as the count of travel nurses employed along with their compensation. Utilizing a color gradient from pink to purple, we portray the dispersion of model runs across time. Purple bands denote distributions that closely align with the median, while pink bands symbolize distributions deviating further from the median. The plot also features blue curves that showcase selected trajectory samples. Notably, calibration targets are marked as black points on the plot. This representation provides a comprehensive insight into the calibration results. Upon initial visual inspection of Figure 4, the calibrated set of runs appears to exhibit stronger agreement with the data for travel nurses in comparison to staff hospital nurses. Nevertheless, it is crucial to highlight that the *y*-axis ranges for staff nurses employed and their wages display a relatively narrow span. This observation underscores the success of our calibration procedure.

## 6 Discussion

### 6.1 Limitations and future directions

Our model overlooks many complexities and factors inherent to the nurse supply and retention issue and there are many other simplifications and limitations with our model. Several limitations and simplifications include

1. The model treats the nursing workforce as a homogeneous population, without stratification by age, years of experience, family status, or other demographic characteristics that are known to influence both wage preferences and pandemic-related work burden. For example, older nurses approaching retirement may respond differently to pandemic-induced work conditions than younger nurses with dependent care responsibilities, and the pandemic-related attrition documented in surveys disproportionately affected nurses in mid-career (58, 61). Age stratification in particular would allow the model to capture the accelerated retirement dynamic observed during COVID-19 surges, which has implications for the long-term contraction of the qualified nurse pool. Incorporating demographic heterogeneity would also allow the supply S-surface to evolve over time as the demographic composition of the workforce shifts, an effect the current model cannot represent;
2. The model was calibrated using national-level data, which excludes smaller regions and travel nurse movement, and therefore obscures the substantial geographic variation in nurse labor market conditions during the pandemic. Simulating regions together would enable exploring worker redistribution policies. COVID-19 hospitalization surges were highly heterogeneous across regions, and access to travel nurses differed markedly between urban and rural hospitals (53). A regional extension of this model, in which separate labor market units are linked through a shared national travel nurse reservoir, would enable exploration of workforce redistribution policies and inter-regional competition dynamics. A precedent for this adaptation exists in a subsequent application of this framework to Virginia’s healthcare workforce, where the model was calibrated to eight distinct regional labor markets using Virginia-specific wage and employment data (59);
3. The present model treats epidemiological dynamics as exogenous, with COVID-19 hospitalizations, cases, and deaths entering as fixed historical time-series inputs rather than being simulated endogenously. This unidirectional coupling, from epidemiology to labor market behavior, is a deliberate simplification that omits the feedback pathway by which nurse supply shortages influence pandemic mortality outcomes and, through them, future epidemiological trajectories (3, 4, 33). Establishing this bidirectional coupling is a goal of future extensions of the framework. However, the unidirectional approach adopted here is not merely a shortcut, but a principled calibration strategy. Fully coupled epi-economic models are substantially harder to calibrate than unidirectional counterparts, because parameter uncertainty in both components interacts and compounds. A staged approach, in which the epidemiological model is first calibrated independently and the strength of behavioral feedback into the epidemiological component is then increased gradually, allows the modeler to preserve the calibrated properties of each component while moving toward full bidirectional coupling. This approach reduces the risk that joint optimization becomes ill conditioned and provides a systematic pathway toward the fully integrated model described in the Introduction.
4. We acknowledge that fatigue, mental health, and risk perception are multifaceted constructs that cannot be fully captured by a single quantitative update rule. Our behavioral model employs exponential discounting via an EWMA process to represent how nurses’ past experiences shape their current perceived work burden. Our use of EWMA and related discounting functions is intended as a parsimonious behavioral abstraction that captures recency-weighted updating in a computationally tractable way, rather than as a complete psychological theory (62, 63).
  This formulation is consistent with adaptive expectations frameworks in labor economics (44). However, exponential discounting implies a constant rate of memory decay, which may not fully capture the psychological reality of nurses’ decision-making. Hyperbolic discounting models—in which the weight placed on past experiences decays more slowly for recent events and more rapidly for distant ones—may better reflect evidence from behavioral economics that salient or traumatic past experiences retain disproportionate influence over time (28, 62, 64–66). Exploring hyperbolic and other non-exponential discounting formulations is a natural direction for future refinements of the behavioral component;
5. A formal sensitivity analysis of the behavioral parameters was not conducted within the scope of the present study. This represents a limitation, as the relative influence of these parameters on model outputs is not fully characterized here. However, a systematic sensitivity analysis was performed in a subsequent application of this modeling framework to Virginia’s healthcare workforce (59), using 40,000 Latin Hypercube sampled case runs. That analysis found that fatigue weighting ($\beta_{F}$), initial nurse demand, and the EWMA discount half-life were the highest-leverage parameters governing work reluctance dynamics, while wage trajectory was primarily sensitive to wage growth rate assumptions and initial demand. Supply dynamics were identified as a mediating output influenced by a diverse range of inputs. These findings provide empirical grounding for the parameter choices made in the present study and suggest that the qualitative conclusions regarding pandemic-induced supply disruption are robust to parameter variation within plausible ranges. A dedicated sensitivity analysis of the national-level model presented here, potentially using the IMABC posterior as a basis for parametric perturbation, remains a priority for future work.
6. While the model serves as an initial exploratory demonstration, further validation and calibration are essential for improved accuracy. Accordingly, its quantitative outputs should be interpreted as illustrative of plausible dynamic ranges rather than as point predictions. Future work should therefore focus on fitting the model to empirical datasets, comparing alternative behavioral specifications, and testing whether the proposed dynamics reproduce observed patterns of response under changing risk, uncertainty, and policy incentives. Such validation would help determine which assumptions are most defensible in specific applications;
7. Despite its mathematical complexity, the model maintains a level of parsimony. It is not intended for forecasting but aims to offer insights into possible dynamics and scenario comparisons. More broadly, the model can be embedded within a robust decision-making or decision-under-deep-uncertainty framework. In these settings, the objective is not to identify a single optimal policy under a known probability model, but rather to compare policies across a wide range of plausible futures and behavioral responses. Our model is well suited to this purpose because it can generate internally consistent trajectories under alternative assumptions about discounting, memory, and behavioral updating, thereby supporting comparative analysis of policy performance across uncertain conditions (67–71). Importantly, robustness evaluation should extend beyond parametric uncertainty to encompass structural uncertainty, including alternative behavioral specifications, functional forms for the supply response surface, and discounting mechanisms, so that policy conclusions can be identified as performing well not merely under calibrated parameter values but across a broader range of plausible model structures and futures (69, 70).

Future extensions can further leverage additional epidemiological indicators as proxies for barriers. For instance, the stringency of Non-Pharmaceutical Interventions (NPIs) can serve as a measure of the challenges in commuting due to personal commitments, with state-level NPI data maintained by Oxford University and Boston University (55, 56). Additionally, the behavioral model parameters that include the weighting of fatigue, distress, and disengagement components, can be further refined through analyses of existing nurse surveys (58, 61, 72), which offer aggregate-level insights into how different pandemic-related factors influence employment decisions.

Moreover, our models detailing hospital decisions and preferences in balancing nurses and travel nurses can gain insights from hospital-level datasets providing utilization, revenues, costs, and charges. These datasets are accessible through the Hospital Cost Reports from the Healthcare Cost Report Information System (HCRIS). RAND researchers have already used HCRIS to explore the relationship between pandemic relief funds and hospital characteristics (73). Incorporating such analyses can contribute to a more refined conceptual model.

### 6.2 Conclusions

We have developed a preliminary economic model to depict hospital nurses’ supply and wage dynamics during a pandemic. The model utilizes sets of ordinary differential equations to capture labor market responses, considering factors like COVID-19 cases and hospitalizations. It incorporates behavioral changes in nurses’ work burden due to risk perceptions, fatigue, and mental distress. Additionally, hospitalization dynamics influence nurse wages and labor costs. Posterior credible intervals for staff and travel nurses’ wages and employment were obtained using an IMABC approach. This conceptual model demonstrates that it is possible to model labor market dynamics as a function of pandemic dynamics, and the model replicates the dynamics observed for workforce participation and wages.

Our model specifically addresses the short-term dynamics or transient effects caused by the pandemic, with a primary focus on retention rather than recruitment, expansion, or other long-term period effects. While recognizing the importance of long-term effects, including expansion, recruitment, and contractions, our focus was on understanding the immediate impacts and dynamics resulting from the pandemic, capturing short-term fluctuations and changes in response to pandemic-induced challenges and uncertainties.

The model’s scenario analyses carry direct implications for the design of pandemic relief programs and hospital staffing policy. The comparison between constrained and unconstrained spending scenarios, governed by the parameter $\lambda_{C}$, illustrates a fundamental tension in relief fund design. Unrestricted reimbursement of travel nurse labor costs reduces the immediate staffing gap but may incentivize excessive reliance on expensive contract labor, driving up total healthcare labor expenditures without improving long-term workforce stability. Conversely, overly stringent relief fund requirements may deter hospitals, particularly rural and safety net facilities with less administrative capacity, from applying for funds they legitimately need (73).

These findings suggest that relief programs are most effective when they are designed to support both immediate staffing needs and durable retention measures, such as wage stabilization for permanent staff, workload reduction initiatives, and investments in the nursing education pipeline. The FEMA Public Assistance program distributed $24.5 billion to hospitals during the COVID-19 pandemic (59), yet the extent to which these funds were allocated in ways that strengthened long-term workforce resilience, rather than simply subsidizing short-term contract labor, remains an open empirical question that integrated models of the type developed here are well positioned to address in future work.

A further long-term consequence of pandemic-era staffing patterns concerns workforce stability. Sustained reliance on travel nurses during repeated surge periods may erode the retention of permanent staff by widening the compensation differential between travel and staff nurses, generating frustration among staff nurses who bear equivalent workloads at lower pay (53, 74). Over time, this dynamic may create a reinforcing cycle in which staff nurse attrition increases reliance on travel nurses, which in turn raises labor costs and further constrains hospitals’ ability to offer competitive wages to permanent staff.

Additionally, pandemic-induced burnout and the accelerated retirement of experienced nurses documented during COVID-19 (58, 61) represent a contraction of the qualified nurse pool that operates on a longer timescale than the short-term supply disruptions modeled here. Future extensions of this framework that incorporate a dynamic qualified pool, allowing the pool to contract in response to sustained high work burden and expand through recruitment and training pipeline effects, would be better positioned to capture these long-term structural consequences.

## Data Availability

The Securities and Exchange Commission disclaims responsibility for any private publication or statement of any SEC employee or Commissioner. This article expresses the author's views and does not necessarily reflect those of the Commission, the Commissioners, or other members of the staff.

## Data availability statement

The raw data supporting the conclusions of this article will be made available by the authors, without undue reservation.
